# Assessment of the pollution and ecological risk of lead and cadmium in soils

**DOI:** 10.1007/s10653-018-0100-5

**Published:** 2018-03-27

**Authors:** Jerzy Wieczorek, Agnieszka Baran, Krzysztof Urbański, Ryszard Mazurek, Agnieszka Klimowicz-Pawlas

**Affiliations:** 1 Department of Agricultural and Environmental Chemistry, University of Agriculture in Krakow, Al. Mickiewicza 21, 31-120 Krakow, Poland; 2 Department of Environmental Management and Protection, Faculty of Mining Surveying and Environment Engineering, AGH University of Science and Technology, al. Mickiewicza 30, 30-059 Krakow, Poland; 3 Department of Soil Science and Soil Protection, University of Agriculture in Krakow, al. Mickiewicza 21, 31-120 Krakow, Poland; 4 Department of Soil Science Erosion and Land Protection, Institute of Soil Science and Plant Cultivation – State Research Institute, ul. Czartoryskich 8, Pulawy, Poland

**Keywords:** Lead, Cadmium, Soil factors, Bioavailability, Risk assessment, Geostatistics

## Abstract

The aim of the study was to assess the content, distribution, soil binding capacity, and ecological risk of cadmium and lead in the soils of Malopolska (South Poland). The investigation of 320 soil samples from differently used land (grassland, arable land, forest, wasteland) revealed a very high variation in the metal content in the soils. The pollution of soils with cadmium and lead is moderate. Generally, a point source of lead and cadmium pollution was noted in the study area. The highest content of cadmium and lead was found in the northwestern part of the area—the industrial zones (mining and metallurgical activity). These findings are confirmed by the arrangement of semivariogram surfaces and bivariate Moran’s correlation coefficients. Among the different types of land use, forest soils had by far the highest mean content of bioavailable forms of both metals. The results showed a higher soil binding capacity for lead than for cadmium. However, for both metals, extremely high (class 5) accumulation capacities were dominant. Based on the results, the investigated soils had a low (Pb) and moderate (Cd) ecological risk on living components. Soil properties, such as organic C, pH, sand, silt, and clay content, correlated with the content of total and bioavailable forms of metals in the soils. The correlations, despite being statistically significant, were characterized by very low values of correlation coefficient (*r* = 0.12–0.20, at *p* ≤ 0.05). Therefore, the obtained data do not allow to define any conclusions as to the relationships between these soil properties. However, it must be highlighted that there was a very strong positive correlation between the total content of cadmium and lead and their bioavailable forms in the soils.

## Introduction

Soil can accumulate heavy metals coming from both natural and a wide range of anthropogenic sources (Fifi et al. 2013; Jiao et al. 2015; Elanzer et al. 2015; Nouri and Haddioui 2016). The major sources of soil contamination by metals are metal mining, smelting, energy and fuel production, industrial activities, solid waste disposal, sludge application, vehicular exhaust, and wastewater irrigation (Ahmadipour et al. 2014; Qin et al. 2016; Kowalska et al. 2016; Baran and Antonkiewicz 2017). Total content of metals is a useful indicator used for the assessment of soil contamination. However, it cannot predict the mobility, bioavailability, and toxicity of trace elements (Vaněk et al. 2005; Fifi et al. 2013; Baran et al. 2014; Elanzer et al. 2015). The major mechanisms responsible for the mobility and bioavailability of metals in soils are surface complex formation, ionic exchange, precipitation, and adsorption to the soil solid phase (Puga et al. 2015; Venegas et al. 2015). The behavior of metals in soil may also be controlled by soil properties, such as pH, redox potential, clay minerals, content of organic matter, Fe and Mn oxide, and calcium carbonate (Mazurek et al. 2017). Soil pollution with heavy metals is a serious problem from the point of view of the environment and public health because the heavy metals tend to persist, circulating indefinitely and eventually bioaccumulating throughout the food chain (Afrifa et al. 2015; Solgi and Khodabandelo 2016; Mohseni-Bandpei et al. 2016). Cadmium and lead play a key role in this pollution and therefore must be considered in the ecological risk assessment (ERA) in a soil system. Generally, the results of the ecological risk assessment can reveal the possibility for soil to be contaminated and even for the ecosystem to be harmed by the concerned heavy metals. Moreover, the ERA procedure may lead to a more precise answer than an approach based only on the concentrations of pollutants at the site (Klimkowicz-Pawlas et al. 2012). Result from ERA can be constructive in designing and planning strategic soil management programmes, policies, practices, and guidance. Moreover, Jiao et al. (2015) proved that heavy metal pollution characteristics and ecological risk assessment are the foundation of soil environmental quality assessment.

Taking into consideration the above, the study aimed to assess the ecological risk of Cd and Pd in the soils. The aim was realized on the basis of the analysis of: (1) the spatial distribution and content of the total and bioavailable forms of lead and cadmium in the soils; (2) the soil lead- and cadmium-binding capacity; and (3) the relationship between Pb and Cd and soil properties using PCA analysis.

## Materials and methods

### Study area and sample collection

**Fig. 1:**
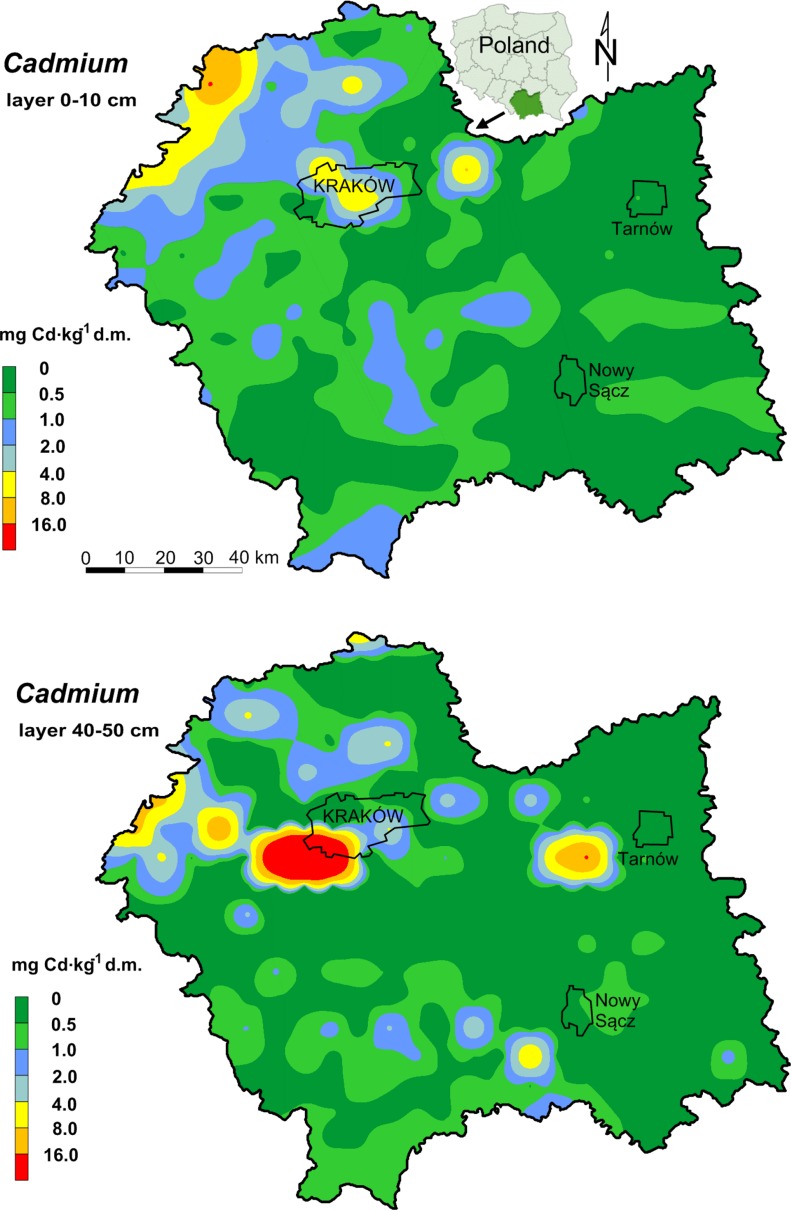
Spatial distribution of Cd in soils

The study was conducted in southern Poland, in the Malopolska province (Fig. 1). A detailed description of the study area and a collection of the sample set were provided in the studies of Baran et al. (2017), and Baran and Wieczorek (2015). Industrial plants, transportation, the power industry, and burning coal in individual home furnaces are the main sources of heavy metals. The second factor, which affects the heavy metal content in soils, is the neighboring Upper Silesian Industrial Basin from the west. The important source of heavy metals in the northwestern part of the area is the mining and metallurgical activity involving the processing of zinc and lead ores (Cabała and Teper 2007; Cabała et al. 2008; Baran and Wieczorek 2015; Baran et al. 2017).

The sampling points were systematically set, based on a 7.5 km × 7.5 km regular grid, with the use of a GPS device (Garmin 62 s, accuracy ± 2 m). In total, 320 grid cells (points) were sampled (Baran et al. 2017). At those points, 5–7 soil subsamples were collected from two depths: 0–10 cm and 40–50 cm using Eijkelkamp samplers and drills. Among the collected soil samples, arable lands constituted 21% (*n* = 66), grasslands—39% (*n* = 126), forests—27% (*n* = 82), and wastelands—13% (*n* = 46). The soil samples were air-dried and sieved through a 2-mm mesh in order to remove large debris, stone, gravel, plant materials, and other materials. A detailed characterization of the basic properties of the soils from the investigated area was provided in the earliest study (Baran et al. 2017). The highest mean organic C content was found in forest soils (7.6%), followed by soils from grasslands (5.5%), wasteland (4.7%), and arable land (2.8%). The soil pH varied from 2.4 to 7.59. Soils with very acid (41%) and acid reaction (29%) were dominant. Silt and clay fractions were dominant in soils from grasslands, arable land, and wasteland. The highest amounts of sand were found in forest soil (Baran et al. 2017).

### Content of lead and cadmium in the soil samples

The total content of lead and cadmium in the soil samples was assessed using microwave digestion with a mixture (1:3 v/v) of ultrapure acids: HCl and HNO_3_. Bioavailable forms of lead and cadmium were extracted with 0.01 mol CaCl_2_ dm^−3^ (Pueyo et al. 2004; Baran et al. 2017). The metal content of these solutions was determined using Perkin-Elmer Optima 7300 DV—an inductively coupled plasma atomic emission spectrophotometer (ICP-AES). The soil samples were analyzed in two replications. The quality of the analysis was verified based on the results of metal determinations obtained on the certified reference material CRM023-050 (Baran et al. 2017). The recoveries for metals ranged from 92 to 102% for Pb and from 90 to 98% for Cd. The precision of the method given as RSD: Cd = 6.9%, Pb = 5.7%.

### Soil lead- and cadmium-binding capacity

The soil lead- and cadmium-binding capacity was assessed using the method developed by Blume and Brummer (1991). This method was used to assess the soil zinc-binding capacity (Wieczorek and Baran 2013). The soil samples were classified into one of the following five classes: class 0—lack of soil lead- and cadmium-binding capacity; class 1—very slight; class 2—slight; class 3—medium; class 4—high; and class 5 indicates soils with an extreme capacity for lead and cadmium accumulation (Baran et al. 2017).

### Potential ecological risk of Pb and Cd in soils

The Potential Ecological Risk Index (PERI) and Hazard Quotient (HQ) were used to determine the ecological risk of cadmium and lead in the soil (Mohseni-Bandpei et al. 2016; Qin et al. 2016; Huang et al. 2017). The PERI for lead and cadmium was calculated based on the following formula (Håkanson 1980)

$$\begin{aligned} \mathop E\nolimits_{\text{r}}^i & = \mathop T\nolimits_{\text{r}}^i \times \mathop C\nolimits_{\text{f}}^i = \mathop T\nolimits_{\text{r}}^i \times \frac{\mathop C\nolimits^i }{\mathop C\nolimits_n^i } \\ PERI & = \mathop {\sum E}\nolimits_{\text{r}}^i \\ \end{aligned}$$

where

- $\mathop E\nolimits_{\text{r}}^i$ is the potential ecological risk of Cd or Pb
- $\mathop T\nolimits_{\text{r}}^i$ is the toxic response factor of Cd, Pb $\mathop T\nolimits_{\text{r}}^i$ = 30 (Cd) and 5 (Pb)
- $\mathop C\nolimits_{\text{f}}^i$ is the index of cadmium and lead pollution
- ${C^{i}}$ represents the measured values of Cd or Pb in the soils
- $\mathop C\nolimits_n^i$ is the background value of Cd or Pb in the study area, $\mathop C\nolimits_n^i$ = 0.22 mg Cd and 18 mg Pb kg^−1^ d.m. (Kabata-Pendias and Pendias 2001)
- PERI is the potential ecological risk by the overall substances.

Studies conducted by different authors have defined four classes of $\mathop C\nolimits_{\text{f}}^i$, five classes of *E*_r_^*i*^, and four classes of PERI (Håkanson 1980). Values of the above parameters and classification are as follows:

- $\mathop C\nolimits_{\text{f}}^i$ < 1 low; 1 ≤ $\mathop C\nolimits_{\text{f}}^i$ < 3 moderate; 3 ≤ $\mathop C\nolimits_{\text{f}}^i$ < 6 considerable; $\mathop C\nolimits_{\text{f}}^i$ ≥ 6 high level of pollution,
- *E*_r_ < 40 low; 40 ≤ *E*_r_ < 80 moderate; 80 ≤ *E*_r_ < 160 considerable; 160 ≤ *E*_r_ < 320—high; *E*_r_ ≥ 320 very high risk,
- PERI < 65 low; 65 ≤ PERI < 130 moderate; 130 ≤ PERI < 260 considerable; PERI ≥ 260 very high risk.

Hazard Quotient for Pb and Cd was calculated by the following equation (Swartjes et al. 2008):

$$HQ = \frac{{\mathop C\nolimits_{\text{e}} }}{{{C_{\text{b}}}}},$$

where

- *C*_e_ is the content of Cd or Pb in the soil (exposure content)
- *C*_b_ is the benchmark value of Cd or Pb (Journal of Laws 2016).

The Hazard Quotient was assessed based on the value of HQ > 1: Potential negative effects relative to the ecological receptors were observed, HQ < 1: Lack of potential negative effects to the ecological receptors was observed (Swartjes et al. 2008; Klimkowicz-Pawlas et al. 2012).

### Geostatistical and statistical analysis

Descriptive statistic variables including mean, median, standard deviation, minimum, maximum, coefficient of variation (CV%) and Pearson’s correlation matrix, and principal component analysis (PCA) were calculated using the Statistica 12 software. The differences between the means were detected by ANOVA and Tukey’s test at a significance level of 0.05. The variability coefficient was compared to the limit values (Wilding 1985; Mucha and Wasilewska-Błaszczyk 2015). The global Moran’s autocorrelation coefficient was calculated for the lead and cadmium content in soils of the Malopolska province. The “queen weight” matrix method was used in the calculations. Values of the univariate local indicator of spatial autocorrelation (LISA) were evaluated. Bivariate Moran’s correlation coefficients were calculated as the dependence between the point location and the spatial weight of the heavy metal content. The significance of Moran’s autocorrelation coefficients and correlation coefficients was evaluated on the basis of random permutation and a comparison with pseudo-p values (Anselin 1995). Empirical semivariograms—the main tool in spatial variability estimation—were created for the content of lead and cadmium in soils of the Malopolska province. Variability maps (surface semivariograms) were elaborated on the basis of omnidirectional semivariograms. Surface semivariograms are helpful in the determination of the directions of the highest and lowest spatial variability of anisotropic soil properties. Variability maps were created using Surfer 8.0 software. Global and local Moran’s autocorrelation coefficients and bivariate correlation coefficients were elaborated using the GeoDa 1.4.6. software.

## Results and discussion

### Content and spatial distribution of lead and cadmium in the soils

**Fig. 2:**
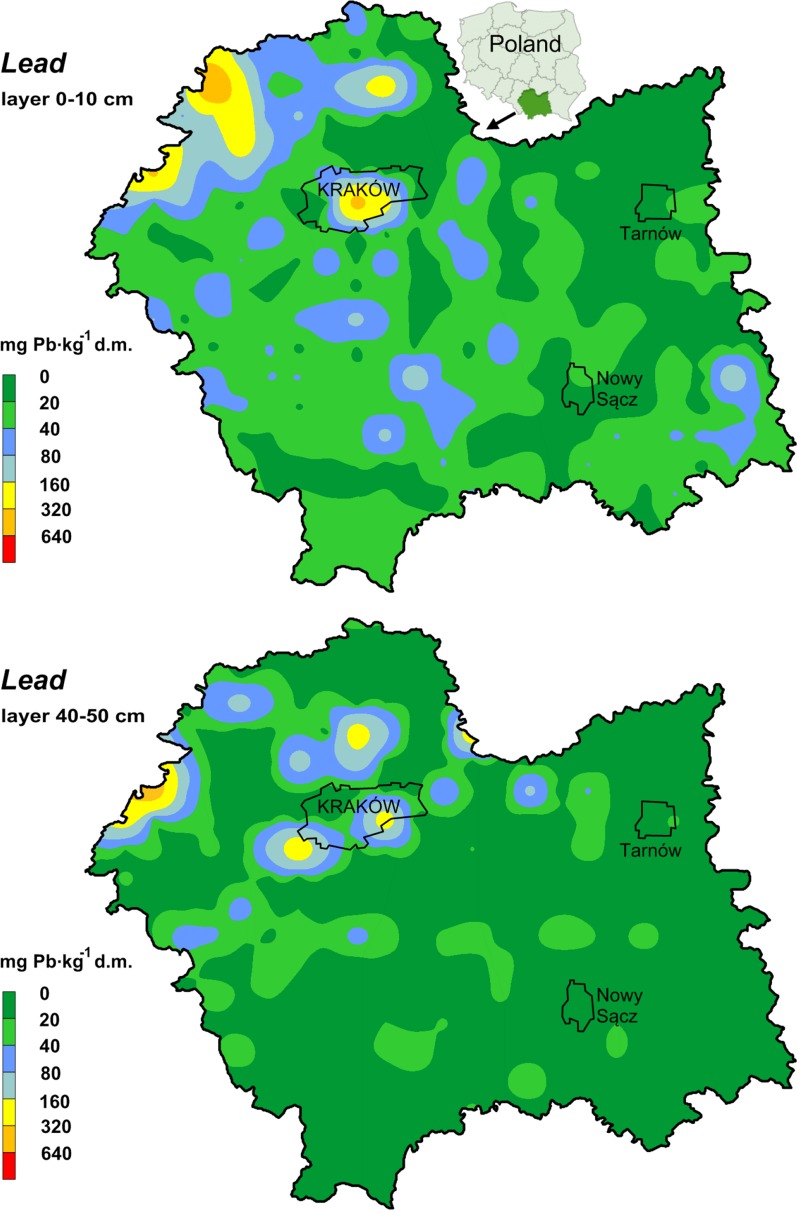
Spatial distribution of Pb in soils

The total content of lead and cadmium in the soil samples is presented in Table 1. The spatial distributions of lead and cadmium in soil are shown separately in Figs. 1 and 2. The content of metals in the topsoil varied from 0.01 to 16.9 mg Cd and from 3 to 586 mg Pb kg^−1^ d.m. In the 40–50 cm soil layer, the total cadmium content ranged from nd to 11.3 mg, and lead—from 2.15 to 470 mg kg^−1^ d.m. However, a lower mean content of lead and a higher mean content of cadmium were found in the 40–50 cm soil layer than in the topsoil (0–10 cm), whereas the differences for lead were statistically significant. The observed mean levels of cadmium and lead in the topsoil were higher than the global average of Cd (0.53 mg kg^−1^ d.m.) and Pb (22 mg kg^−1^ d.m.) content in uncontaminated soils (Kabata-Pendias and Pendias 2001). Depending on the type of land use, the mean total cadmium content in the soils formed the following order: grassland > arable land > forest soils > wasteland, and lead: arable land > forest soils > wasteland > grassland (Table 1). However, the study showed no significant differences between the metal content in the soil depending on the type of land use, despite visually high differences (Table 1, Figs. 1, 2).

**Table 1:** Total content of Cd and Pb in the soils (mg kg^−1^ d.m.)

Parameter	Mean^b^	SD	Minimum	Maximum	Median	Skewness	Kurtosis	CV %^a^
Cd (0-10 cm)	0.93a	1.59	0.01	16.9	0.49	5.41	39.1	171
Arable land	0.97a	1.49	0.07	7.75	0.49	3.49	**–**	154
Grassland	0.99a	1.88	0.02	16.9	0.54	6.07	**–**	189
Forest soils	0.88a	1.34	0.02	8.34	0.39	3.38	**–**	152
Wasteland	0.78a	1.25	0.01	8.35	0.48	5.68	**–**	160
Cd (40-50 cm)	1.24a	5.38	0	11.30	0.36	9.69	–	438
Pb (0-10 cm)	38.3a	56.9	3.0	586	22.2	5.45	38.4	148
Arable land	50.2a	89.6	11.3	586	24.0	5.40	**–**	178
Grassland	30.6a	26.4	3.00	148	23.3	5.70	**–**	86
Forest soils	41.5a	61.5	8.00	356	19.4	3.35	**–**	148
Wasteland	36.6a	46.9	6.30	309	23.6	5.74	**–**	128
Pb (40-50 cm)	23.0b	42.2	2.15	470	13.2	6.50	–	183

^a^CV %—variation coefficient

^b^Means followed by the different letters in line indicate significant differences at *α* ≤ 0.05 according to the *t* Tukey test

**Fig. 3:**
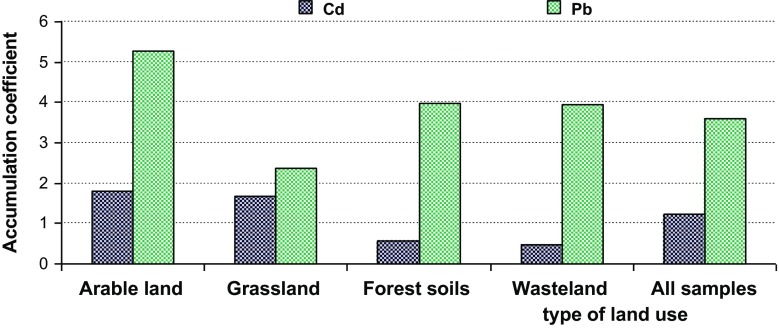
Accumulation coefficient of metals as the content ratio: Cd_(0–10 cm)_/Cd_(40–50 cm)_ and Pb_(0–10 cm)_/Pb_(40–50 cm)_ in soils

Heavy metal content variability should be described as extremely high, which is evident on the basis of variability coefficient that exceeded 140% (Wilding 1985; Mucha and Wasilewska-Błaszczyk 2015). The coefficients of the variation values of heavy metals that are originated from natural sources are relatively low, while CV values of heavy metals affected by anthropogenic sources are quite high (Baran et al. 2017). The total lead and cadmium content exhibited a high degree of variability, indicated by high values of coefficients of variation for Cd—171% (0–10 cm), 438% (40–50 cm), and for Pb—148% (0–10 cm) and 183% (40–50 cm). The characteristic trait of both of heavy metals was positive and very high values of skewness, which was presented by other authors in papers devoted to a similar topic (Imperato et al. 2003; Kabała et al. 2009; Liu et al. 2013). The leptokurtic character of distribution was evaluated for distribution of the studied heavy metals.

**Fig. 4:**
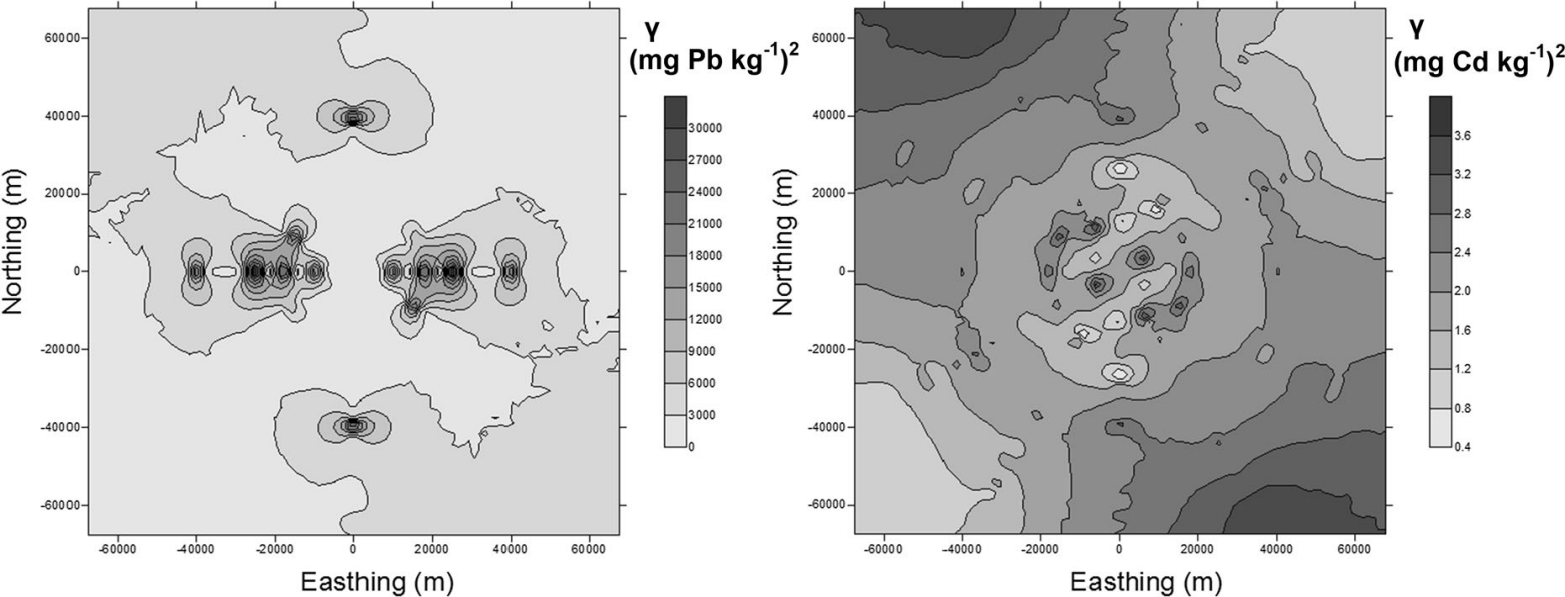
Semivariogram maps (surface semivariograms) created on the basis of the total content of Pb and Cd in soils of the Malopolska district. Values of semivariance (*γ*) of total Cd and Pb are presented on the maps. Semivariogram map is a plot of experimental (directional) semivariogram and coordinates calculated on the basis of point location vertical (Northing) and horizontal (Easting) differences (Mucha and Wasilewska-Blaszczyk 2015; Bartuś 2012). Geometric anisotropy in NW–SE direction shows the highest variation within data

It was stated that univariate Moran’s index calculated for the cadmium and lead content was positive and significant. This is a proof that there are clusters with a high content (northwest part) and a low content (southeast part) of cadmium and lead on the investigated area (Table 2). Simultaneously, a significant dependence between the localization of the sampling points and the accumulation of elements on the basis of bivariate local Moran’s correlation coefficient values was found. In the eastern direction, the content of heavy metals was lower, and in the northern, it was higher. Higher latitude values of the Malopolska province were connected with a higher concentration of cadmium and lead in soil. Higher spatial correlation coefficients were calculated for Cd than for Pb, which proved a stronger relationship between the localization and the cadmium concentration in soil. Maps of trace element variability are presented in the form of surface semivariograms (indicatrix) (Fig. 4). Based on the indicatrix graphs, it can be stated that the highest variability of the studied heavy metals in the Malopolska province is from the northwest to southeast direction. It is worth pointing out that western and central parts (with Krakow) of the area are industrialized, whereas northern and eastern parts of Malopolska typically have a agricultural character. These observations demonstrate that the mining and metallurgical activity, which has been conducted for several hundred years, is an important factor affecting heavy metal content in the soil. Soils in the vicinity of metallurgical industry contain from 20 to 4705 mg Pb kg^−1^ and from 3 to 67.5 mg Cd kg^−1^ (Vaněk et al. 2005) or more than 30000 mg Pb kg^−1^ and 90 mg Cd kg^−1^ (Ettler et al. 2005). The high content of cadmium and lead in the soil samples is connected with the occurrence of metalliferous minerals whose accumulations depend on natural and anthropogenic factors. The most important of these factors are: geological structure and erosion of shallow ore-bearing Triassic formations; historical mining as well as processing of zinc and lead ores. Above factors are responsible for the surface deposition of waste rich in cadmium and lead; the emission of metal-rich dust from the zinc works; and the high emission of industrial dust from the Upper Silesian Industrial Region as well as the eolian redeposition of zinc–lead–iron minerals from above ground landfills designed for post-flotation and metallurgical waste (Baran et al. 2014). Other authors have also found that soils in areas of zinc–lead ore mining and metallurgy areas have very high levels of lead and cadmium (Cabała et al. 2008; Kowalska et al. 2016).

**Table 2:** Global and local Moran’s autocorrelation indices and Moran’s correlation coefficients

Dependance		Global autocorrelation index and bivariate correlation index	Local indicator of spatial autocorrelation (LISA)** [%]				
			N	H–H	L–L	L–H	H–L
Cd	lagCd/Cd	0.4283*	70.0	6.88	20.3	1.88	0.94
	lagCd/X	−0.3481*	70.6	0.31	0.94	8.13	20.0
	lagCd/Y	0.3036*	70.9	9.06	10.6	0.00	9.38
Pb	lagPb/Pb	0.3344*	74.4	5.94	16.9	1.88	0.94
	lagPb/X	−0.2902*	75.3	0.00	2.19	7.50	15.0
	lagPb/Y	0.2299*	75.9	7.19	4.38	0.00	12.5

*Significant at pseudo* p* value 0.05, **LISA—local indicator of spatial autocorrelation; *N* without autocorrelation, H–H clusters with high values, L–L clusters with low values, L–H low values are surrounded by high values (“coldspots”), and H–L high values are surrounded by low values (“hot spots”)

Based on the results, the accumulation coefficients of the metals were calculated. The accumulation coefficient was calculated as the content ratio: Cd_(0–10 cm)_/Cd_(40–50 cm)_ and Pb_(0–10 cm)_/Pb_(40–50 cm)_ (Wieczorek and Zadrożny 2013) (Fig. 3). Lead was characterized by higher values of accumulation (from 2.37 to 5.27) than cadmium (from 0.48 to 1.79). It was confirmed that the lead profile distribution shows a decrease with depth, which is in accordance with the studies of other authors (Vaněk et al. 2005). The increased content of cadmium in the deeper layer could be explained by historical contamination originating from long-term mining and smelting activities as well as a partial transfer of cadmium in the soil profile (Kowalska et al. 2016). It may also be associated with a high mobility of cadmium in the soils and it being leached to the lower profiles, leading to a decrease in the accumulation of the metal in the surface layer.

### Assessment of bioavailable forms of Cd and Pb in soils extracted with 0.01 mol CaCl_2_ dm^−3^

**Fig. 5:**
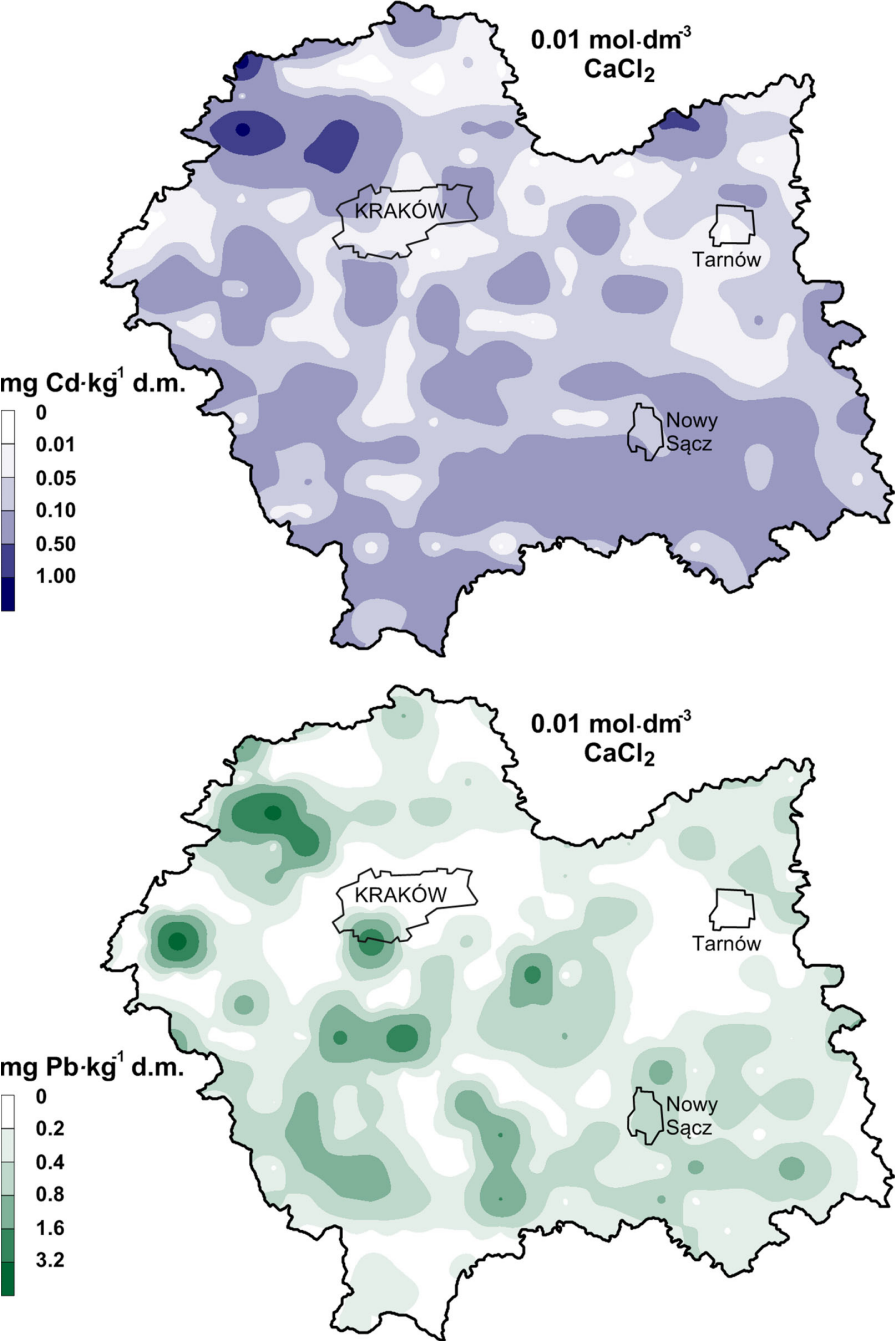
Spatial distribution of bioavailable forms of Cd and Pb in soils

The total content of metals is an important indicator of soil contamination, but it cannot give sufficient information about mobility and toxicity of metals (Baran et al. 2014; Ahmadipour et al. 2014; Kim et al. 2015). In the risk analysis, the knowledge about the content of readily soluble or exchangeable metal forms is particularly useful due to their possible mobilization from the solid phase and moving in the environment, where they become bioavailable (Vaněk et al. 2005). The content of bioavailable forms of cadmium and lead in the soils is presented in Table 3 and Fig. 5. The content of bioavailable forms varied within a wide range from nd to 1.23 mg Cd kg^−1^ d.m. and from nd to 4.73 mg Pb kg^−1^ d.m. Among the different types of land use, forest soils had significantly the highest mean content of bioavailable forms of metals, followed by arable land > grassland > wasteland in the case of Cd, whereas grassland > wasteland > arable land > in the case of Pb. The computed coefficient of variation (CV) for cadmium was between 86% (wasteland) and 131% (forest), and for Pb—between 71% (wasteland) and 148% (grassland) (Table 3). However, the spatial distribution of bioavailable forms of Cd and Pb in the soil was generally similar (Fig. 5). It was demonstrated that heavy metal solubility in 0.01 mol CaCl_2_ · dm^−3^ was very low for Pb and low for Cd. The solubility of the metals in the soils with respect to their total content ranged from 0 to 98% for Cd and Pb, with a mean of 24% (Cd), and from 0 to 32%, with a mean of 1.7% (Pb), respectively (Table 3). Elanzer et al. (2015) proved that heavy metals from anthropogenic sources tend to be more mobile than pedogenic or lithogenic ones. The highest content of bioavailable forms of metals in the soil was also found in the northwestern part of the study area—mining and processing of zinc–lead ores (Fig. 4). However, the solubility (mobility) of metals from soils sampling in this area was generally low or moderate (9–13% for Cd, and 0.6–3% for Pb). The ores can be found in ore-bearing Diplopora dolomites, which are a source not only of lead and cadmium, but also of calcium and magnesium carbonate, which in turn have an alkaline effect on the soil environment and, as consequence, on the environment, a beneficial effect on binding metals into stable carbonate minerals (Cabała et al. 2008; Baran et al. 2014). Among the different types of land use, the highest solubility of Cd was in the forest soils, followed by arable land > grassland > wasteland, whereas the solubility of Pb formed the following order: grassland > forest soils > arable > wasteland land. Our results confirmed the higher mobility of Cd in comparison with Pb. Lead seems to be more stable than cadmium in soil because it is bound stronger to the crystalline structures of the mineral and soil organic matter higher than cadmium (Ahmadipour et al. 2014; Baran et al. 2014). Other studies also have proved that cadmium is very mobile in soil environments and shows potentially high toxicity for living organisms, even at low concentrations (An 2004; Fifi et al. 2013). The ionic strength of calcium chloride is similar to the one of pore water; Ca^2+^ is better able to displace metals from exchange sites than other ions, and low salt concentration reduces analytical interferences (Pueyo et al. 2004; Ettler et al. 2005; Kim et al. 2015). Numerous authors have found that application of 0.01 mol CaCl_2_ dm^−3^ enables separation of a mobile and easily bioavailable heavy metal form from soil, which in natural conditions may become released from soil posing a real threat to living organisms (Pueyo et al. 2004; Meers et al. 2007; Kim et al. 2015). Moreover, the content of metals in soils, extracted by 0.01 mol CaCl_2_ dm^−3^, is generally well correlated with the response of living organisms (Meers et al. 2007; Baran et al. 2014; Kim et al. 2015). Properties such as the pH value, organic carbon content, texture, and redox condition are responsible for the bioavailability of metals in soils (Blume and Brummer 1991; Ahmadipour et al. 2014; Baran et al. 2014; Elanzer et al. 2015). It was proved that the solubility of heavy metals increases at a low soil pH and decreases in soils with a high content of organic matter. The highest content of mobile forms of Cd and Pb in the forest soil was found. Moreover, the solubility of Cd was higher in the forest soils than in other soils. Close to 89% of forest soils had very acid and acid reactions as well as a high content of organic carbon and sand. The high content of organic matter in forest soils could have decreased the solubility of lead (Table 3). However, sometimes when the pH was increased above 7.5, the solution concentration of metals increased (Fairbrother et al. 2007). This process is connected with the solubilization of organic complexing ligands, which effectively compete with the soil surfaces for the metal cation. Most functional groups of complexing ligands are weak acids, so the stability of the metal complex is pH dependent with little association in acid media. The degree of association increases with the pH. In soils with significant levels of dissolved organic matter, increasing the soil pH may actually mobilize metal due to complex formation (Fairbrother et al. 2007; Draszewska-Bozan 2017).

**Table 3:** Content of bioavailable forms of Cd and Pb in soils (mg kg^−1^ d.m.) extracted with 0.01 mol CaCl_2_ dm^−3^

Parameter	Mean^b^	SD	Minimum	Maximum	Median	CV %^a^	% Extraction^d^
Cd (all samples)	0.11	0.14	nd^c^	1.23	0.08	125	24 (0–98)
Arable land	0.10a	0.12	nd	0.58	0.07	119	23 (0–94)
Grassland	0.10a	0.09	0.005	0.45	0.07	90	21 (0–97)
Forest soils	0.17b	0.22	nd	1.23	0.10	131	34 (0–98)
Wasteland	0.09a	0.08	nd	0.36	0.07	86	18 (1–61)
Pb (all samples)	0.410	0.52	nd	4.73	0.24	127	1.70 (0–32)
Arable land	0.25a	0.18	0.04	0.81	0.18	75	1.46 (0–10)
Grassland	0.34a	0.50	nd	4.73	0.21	148	1.83 (0–32)
Forest soils	0.74b	0.71	nd	4.08	0.52	97	1.68 (0–5)
Wasteland	0.29a	0.21	0.05	0.85	0.19	71	1.64 (0–9)

^a^CV %—variation coefficient

^b^Means followed by the different letters in line indicate significant differences at *α* ≤ 0.05 according to the *t* Tukey test

^c^nd—not detected

^d^Percentage of bioavailable fraction in relation to total content

### Soil cadmium- and lead-binding capacity

**Fig. 6:**
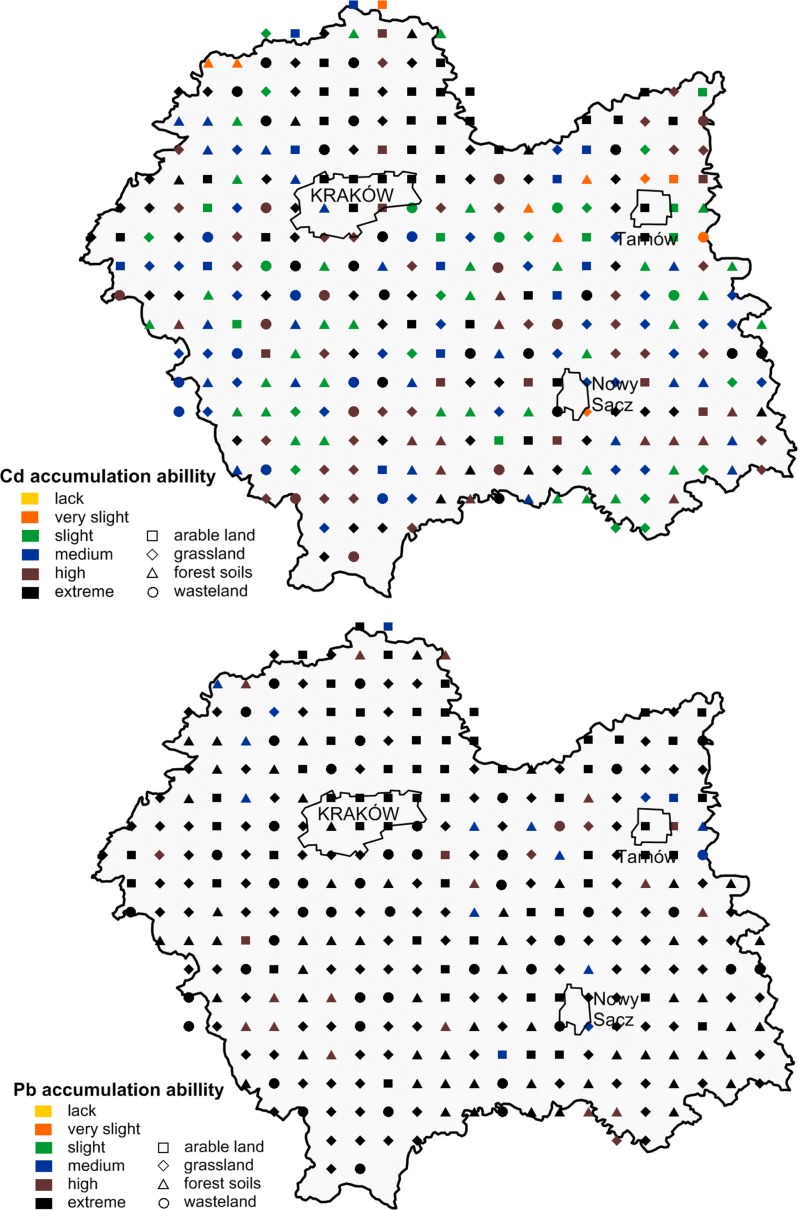
Soil cadmium- and lead-binding ability

The soil cadmium- and lead-binding capacity is presented in Fig. 6. These results show higher soil binding capacity for lead than cadmium. We found that 88% of soil samples had an extreme (class 5) lead accumulation capacity. Soil with a very high (class 4) lead-binding capacity constituted 7% of all the soil samples, whereas that with medium (class 3) constituted only 5%. For cadmium, soils with an extremely high (class 5) Cd-binding capacity were also dominant (constituting 33%). Classes 4, 3, and 2 were represented by 21, 23, and 20% of the soils, respectively. Only 3% of the soil samples had very slight (class 1) cadmium-binding capacity (Fig. 5). Depending on the type of land use, the lead-binding capacity in arable land soils formed the following order: extreme (90%) > high, medium (5% each); in the grassland: extreme (94%) > high (4%) > medium (2%); in the forest soil: extreme (71%) > high (18%) > medium (11%); and in the wasteland: extreme (96%) > high, medium (2% each). Soil binding capacity for cadmium was more different and dependent on the type of land use: extreme (52%) > medium (18%) > high (15%) > slight (12%) > very slight (3%)—arable soils; slight (43%) > medium (27%) > high (15%) > extreme (10%) > very slight (6%)—forest soil; extreme (41%) > high (26%) > medium (20%) > slight (11%) > very slight (3%)—wasteland; and extreme (34%) > high (27%) > medium (25%) > slight (12%) > very slight (2%)—grassland. The main reason for the slight cadmium-binding capacity in the forest soil was associated with acid sandy soils. Towers and Paterson (1997) as well as Wieczorek and Baran (2013) also indicated that the soil pH is the key factor controlling the metal-binding capacity of the soil; however, organic C and clay content affect the final classification.

### Assessment of the potential ecological risk of cadmium and lead in soils

The calculated values of the potential ecological risk factors $\mathop E\nolimits_{\text{r}}^i$ for each metal, PERI for both metals, and hazard quotient for cadmium and lead is presented in Table 4. The mean values of *C*_f_ for cadmium and lead generally exceeded 1. However, higher values of this index were shown for Cd than Pb. Index of cadmium pollution (*C*_f_) indicated that soils were moderately (48% of the samples), considerably (26% of the samples), lowly (15% of the samples), and highly (11% of the samples) contaminated with cadmium. It was found that 51% of soil samples were moderately, 36% lowly, 8% considerably, and only 5% highly contaminated with lead. The highest share of soils highly polluted with cadmium and lead was found in forest areas—16% and 6% of the samples, respectively. The potential ecological risk index for individual elements ($\mathop E\nolimits_{\text{r}}^i$) calculated for cadmium ranged from 1.56 to 2303 with a mean of 126, and for lead, it ranged from 0.82 to 162 with a mean of 10.6 (Table 4). The results of $\mathop E\nolimits_{\text{r}}^i$ indicated that Pb had a low risk (96% of the samples) to the local ecosystem, while Cd reported moderate (35% of the samples), considerable (25% of sample), low (23% of the samples), and high and very high risk (7 and 8% of the samples). The highest share of soils with high and very high risk of cadmium was found in grassland. PERI for the metals ranged from 4.28 to 2466 with a mean of 137 (Table 4). PERI results showed that: 42% of the samples had low risk; 33% of the samples were with moderate; 15% of the samples were with severe; and 9% of the samples had a serious metal risk. The potential ecological risk factors $\mathop E\nolimits_{\text{r}}^i$ calculations for zinc showed that 318 samples had a low potential ecological risk to the environment (Baran et al. 2017). In the Malopolska area, the ecological risk is connected mainly with the soil pollution with Cd. The same results were found in the study of Elanzer et al. (2015). The HQ values for cadmium were between 0.01 and 5.63, and for Pb—between 0.01 and 2.35 (Table 4). Only in 4% (Cd) and 1% (Pb) of the sampling points, HQ values indicated a potential harmful effect on ecological receptors. Depending on the type of land use, the HQ for Cd in the soils formed the following series: grassland ≈ arable land > wasteland > forest soils, and for Pb: grassland > arable land > wasteland ≈ forest soils (Table 5). The highest values of $\mathop E\nolimits_{\text{r}}^i$, PERI, and HQ for metals were found in the soil of the northwestern part of the Malopolska province. Our previous studies also found high values of the geoaccumulation index (*I*_geo_), pollution index (PI), and integrated pollution index (IPI) for heavy metals in soils of this region (Baran and Wieczorek 2015; Baran et al. 2017). However, it should be highlighted that some soils in northwestern Malopolska have a naturally high content of cadmium and lead because these soils were formed from bedrocks containing considerable amounts of these metals (Cabała and Teper 2007; Baran et al. 2017).

**Table 4:** Statistical results of potential ecological risk index and hazard quotient of Cd and Pb

Elements	Risk	Arable land	Grassland	Forest soils	Wasteland	All samples
Cadmium	*C*_f_	4.40 (0.32–35.2)^a^	4.0 (0.11–76.7)	4.0 (0.11–37.9)	3.55 (0.05–39.1)	4.22 (0.05–6.8)
	$\mathop E\nolimits_{\text{r}}^i$	132 (9.55–1057)	135 (3.27–2303)	120 (3.36–1138)	106 (1.56–1170)	126 (1.56–2303)
	HQ	0.32 (0.02–2.58)	0.33 (0.01–5.63)	0.09 (0–0.83)	0.25 (0.05–2.86)	0.26 (0.01–5.63)
Lead	*C*_f_	1.84 (0.41–23.4)	2.06 (0.16–32.6)	2.85 (0.60–17.8)	1.45 (0.30–11.87)	2.13 (0.16–32.5)
	$\mathop E\nolimits_{\text{r}}^i$	9.19 (2.03–117)	10.3 (0.82 –162)	14.3 (2.99–85.9)	7.23 (1.52–59.3)	10.6 (0.82–162)
	HQ	0.13 (0.03–1.69)	0.15(0.01–2.35)	0.10 (0.02–0.62)	0.10 (0.02–0.85)	0.13 (0.01–2.35)
PERI		141 (12.1–1150)	145 (5.53–2466)	134 (12.2–1229)	113 (4.28–129)	137 (4.28–2466)

^a^Mean value, range

**Table 5:** Relationships between soil properties and concentration of cadmium and lead in soils

Parameters	C—org.	Sand	Silt	Clay	Cd Total	Pb Total	Cd CaCl_2_	Pb CaCl_2_
Sand	0.06							
Silt	−0.12*	−0.78*						
Clay	0.03	−0.78*	0.23*					
Cd Total	0.17*	0.25*	−0.21*	−0.18*				
Pb Total	0.29*	0.26*	−0.21*	−0.20*	0.79*			
Cd CaCl_2_	0.20*	0.07	−0.05	−0.06	0.14*	0.21*		
Pb CaCl_2_	0.22*	0.12*	−0.07	−0.12*	0.02	0.18*	0.32*	
pH	−0.15*	0.06	0.03	−0.10	0.11	0.07	−0.10	−0.11*

*Significant at *p* ≤ 0.05

### Correlation analysis and PCA analyses

**Fig. 7:**
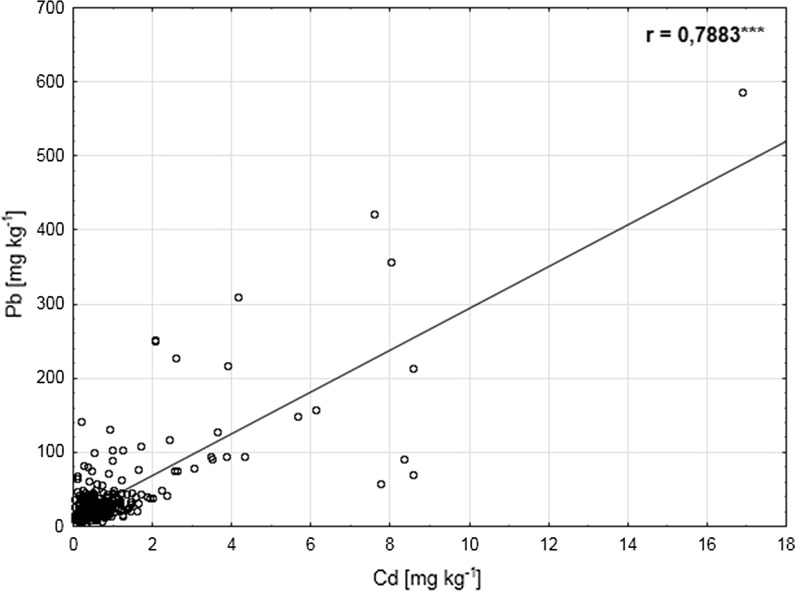
Scatter plot showing dependence between Cd and Pb content in studied soils

The correlation analysis is presented in Table 5. It is generally believed that larger numbers of samples allow to detect significant relations even with low values of the correlation coefficient. However, even statistically significant correlations, when they have low values, do not allow to create good prediction models. The research (*n* = 320) showed a substantial number of statistically significant correlations, for instance, positive correlations for: sand and the content of both metals (total content) and lead content (in calcium chloride), total cadmium content and bioavailable cadmium content, as well as total lead with the content of cadmium and lead forms extracted with CaCl_2_. A positive correlation of organic carbon with the total and bioavailable content of the metals was also recorded. Negative correlations between the silt and clay content and the total content of cadmium and lead, as well as between the pH and the content of clay, organic C and metal forms determined in a calcium chloride extract were also shown. However, all the above-mentioned correlations, despite being statistically significant, are characterized by a very low correlation coefficient oscillating within the ± 0.1–0.3 range. It prevents us from drawing any conclusions from this information as to relationships between these soil properties. It was shown that only a few correlations were simultaneously statistically significant and had values of the coefficient higher than ± 0.7. Those were correlations between the content of sand, silt, and clay and between the total content of cadmium and lead (Fig. 7).

**Fig. 8:**
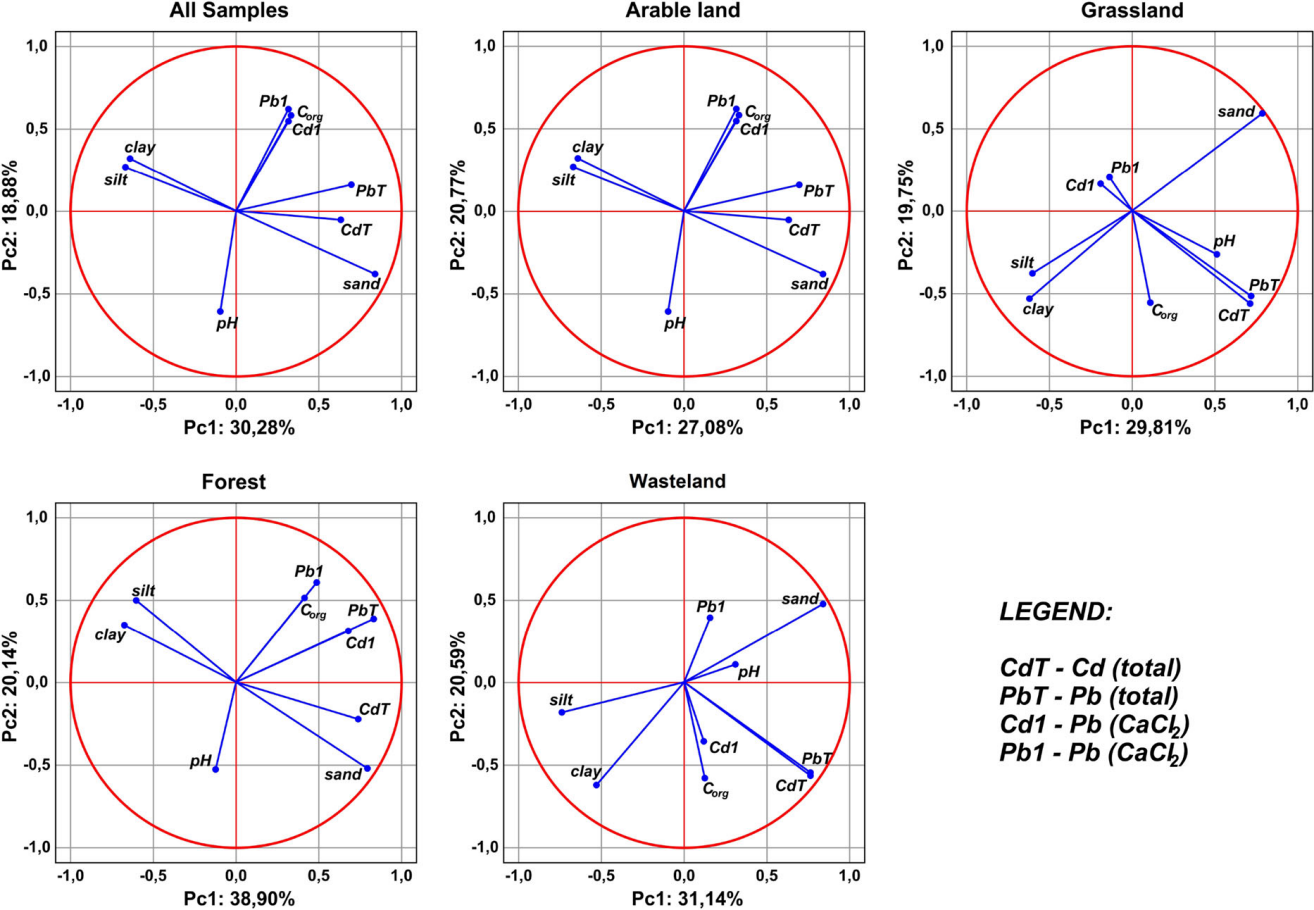
Results of PCA relationships between cadmium and lead and soil factors

Principal component analysis, conducted using centroid method, allowed to observe several interesting relationships between the investigated variables (Table 6, Fig. 8). Firstly, in each case, regardless of the type of land use, none of the parameters was clearly strongly associated with a specific factor. Only in one case (arable lands), the value for sand, as a component parameter of factor no. 1, was more than 0.9 (with negative sign). In other cases, coordinates for individual parameters ranged from ± 0.5 to the maximum of ± 0.83. Coordinates and values of the constituents are represented graphically by sections of individual parameters, usually arranged in diagonals, and rarely according to lines of specific factors (Fig. 8). This translates into the contribution of variables in clarifying the total variance of primary factors. For the first two primary factors, relatively low values of the variance being explained are recorded, where, for forest grounds, the first two factors can explain almost 60% of the total variance (Table 6). For the other forms of land use, it is approximately only 50%. Granulometric fractions (sand, silt, and clay) and the total content of cadmium and lead are the most frequent component of the first primary factor. Exceptions were soils on arable lands, where Pb and Cd (total) constituted the principal component of the second primary factor, and in the case of grasslands and wasteland, they are simultaneously a component of the first and the second primary factor. Centroid graphs also made it possible to observe and define the previously mentioned correlation relations between the investigated parameters. In general, in all the cases, there was a very strong positive correlation between the total content of cadmium and lead and their bioavailable forms, as well as between granulometric fractions. An exception to some of the observed rules is forest soils and wasteland, where a very weak positive correlation between the total content of Pb and Cd and bioavailable forms of the metals (forests) and a negative correlation between cadmium and lead forms determined in CaCl_2_ solution (wasteland) were observed. Moreover, in the case of these forms of land use, a positive correlation between the content of organic C and bioavailable form of lead (forests) and cadmium (wasteland) was shown. At the same time, in almost each case, it is possible to observe on the monoplots that there is practically no relationship between the total content of Pb and Cd and granulometric fractions. A small exception to this rule can be observed in soils of forest grounds and in data for all types of land use (n = 320). Lack of a clear repeatability in monoplots generated for the entire set of soil samples (n = 320) as well as for individual forms of land use induces to formulation of the assumption that it is the form of land use and location of sample collection that have an effect on distribution of individual soil parameters (including availability and metal content).

**Table 6:** Component matrix for variables

Variables	All samples		Arable land		Grassland		Forest		Wasteland	
	PCA 1	PCA 2	PCA 1	PCA 2	PCA 1	PCA 2	PCA 1	PCA 2	PCA 1	PCA 2
pH	−0.0943	−0.5966	0.1181	−0.3306	0.5083	−0.2592	−0.1229	−0.5225	0.3077	0.1092
C—org.	0.3301	0.5715	−0.051	−0.4038	0.1118	−0.5508	0.4106	0.5084	0.1249	−0.5743
Sand	0.8313	−0.3753	−0.9316	0.3036	0.7882	0.5933	0.7856	−0.5136	0.8313	0.4738
Silt	−0.663	0.2705	0.743	−0.0832	−0.5948	−0.3752	−0.5984	0.4898	−0.7331	−0.1781
Clay	−0.6381	0.3169	0.7134	−0.4137	−0.6098	−0.524	−0.6676	0.3477	−0.5234	−0.616
Cd_Total_	0.6282	−0.0467	−0.3722	−0.8342	0.7109	−0.5537	0.7303	−0.2199	0.7628	−0.55
Pb_Total_	0.6894	0.162	−0.4345	−0.7834	0.7128	−0.5132	0.825	0.3855	0.7544	−0.5431
Cd_CaCl2_	0.3144	0.5458	0.3981	−0.0057	−0.1868	0.1649	0.671	0.3093	0.1169	−0.3531
Pb_CaCl2_	0.3175	0.613	0.0747	0.1294	−0.1305	0.2048	0.4822	0.6031	0.1579	0.3914
% of the total variance	30.28	18.88	27.08	20.77	29.81	19.75	38.90	20.14	31.14	20.59

## Conclusions

The content, distribution, pollution, soil binding capacity, and ecological risk assessment of cadmium and lead in soils of Malopolska were investigated in this study. The investigation of 320 soil samples from the differently used areas (grassland, arable land, forest, wasteland) revealed very high variation in the metal content in the soils. The high variation in the metal content in the soils was connected with the occurrence of metalliferous minerals in the northwestern part of the study area whose accumulations depend on both natural and anthropogenic factors. However, the pollution of Malopolska soils with cadmium and lead is moderate. Generally, a point source of lead and cadmium pollution was noted in the study area. The highest content of cadmium and lead was found in the north-western part of the study area—the industrial zones (mining and metallurgical activity). These findings are confirmed by the arrangement of semivariogram surfaces and bivariate Moran’s correlation coefficients. Among the different types of land use, forest soils had significantly the highest mean content of bioavailable forms of both metals. We found higher soil binding capacity for lead than cadmium. However, for both metals, extremely high (class 5) accumulation capacities were dominant. Based on the results of ecological risk assessment, the investigated soils had low (Pb) and moderate (Cd) ecological risk on the live ecosystem. Analysis of potential ecological risk showed an uneven distribution of ecological risk in the study area. However, only 9% of the soils indicated serious metal risks to ecological receptors. According to our results, soil properties such as organic C, pH, sand, silt, and clay content correlated with the content of total and bioavailable forms of metals in the soils. The obtained correlations, despite being statistically significant, were characterized by very low values of the correlation coefficient. Therefore, the obtained data do not allow to define any conclusions as to the relationships between these soil properties. However, it must be highlighted that there was a very strong positive correlation between the total content of cadmium and lead and their bioavailable forms in the analyzed soils.

This study presents important information about soil lead and cadmium content and provides sufficient methods for assessment of contamination with these metals and identification of soil properties which affect the behavior of the metals in soils. Moreover, the study of total and bioavailable forms of cadmium and lead content as well as metal-binding capacity is an important element to evaluate the risk of transfer of both metals in the soil–plant–human chain. However, a division of the study area based on human health risk should be considered a research focus.
